# Determinants of speech and language delay among children aged 12 months to 12 years at Yekatit 12 Hospital, Addis Ababa, Ethiopia: a case–control study

**DOI:** 10.1186/s12887-024-04862-4

**Published:** 2024-06-12

**Authors:** Feven Y. Moges, Zuriyash Mengistu, Sosina W. Tilahun

**Affiliations:** 1 Department of Nursing, College of Health Sciences, Arsi University, Asella, Ethiopia; 2 Department of Nursing, College of Health Sciences, Addis Ababa University, Addis Ababa, Ethiopia

**Keywords:** Speech and language delay, Children, Yekatit 12 Hospital, Addis Ababa, Ethiopia

## Abstract

**Background:**

Speech and language delay among children can result in social interaction problems, attention difficulties, decreased writing and reading abilities, and poor cognitive and behavioral development. Despite the mounting prevalence of speech and language delays in Ethiopia, there is a lack of literature addressing the factors contributing to this delay. Consequently, this study aims to identify determinants of speech and language delay among children aged 12 months to 12 years at Yekatit 12 Hospital in Addis Ababa, Ethiopia.

**Methods:**

We conducted an institutional-based at Yekatit 12 Hospital, unmatched case–control study with 50 cases and 100 controls aged 12 months to 12 years. Interviewer-administered questionnaires were used to collect data from the parents or caregivers of the participating children. Epi Info v7 was used for sample calculation, and SPSS v26 was used for analysis. The chi-square test was performed to determine the relationship between speech and language delay and determining factors, which was then followed by logistic regression. The significant determining factors were identified based on the adjusted odds ratio (AOR), with a 95% CI and *p*-value (< 0.05).

**Results:**

Case group constituted 23 males and 27 females, totaling 50 children. Upon completing the multivariate analysis, birth asphyxia [AOR = 4.58, 95CI (1.23–16.99)], bottle-feeding [AOR = 4.54, 95CI (1.29–16.04)], mother–child separation [AOR = 2.6, 95CI (1.05–6.43)], multilingual family [AOR = 2.31, 95CI (1.03–5.18)], and screen time greater than two hours [AOR = 3.06, 95CI (1.29–7.28)] were found to be statistically significant determinants of speech and language delay.

**Conclusions:**

Our study found that birth asphyxia, bottle-feeding, mother–child separation, being from a multilingual family, and excessive screen time contribute significantly to speech and language delay. As a result, it is important to develop interventions that target these modifiable factors, while also ensuring that early diagnosis and treatment options are readily accessible.

## Background

Speech and language are crucial for communication, allowing us to express thoughts and emotions [1]. Language organizes concepts, while speech conveys these concepts audibly [2–5]. Development of speech and language begins early in childhood and progresses as we age [6]. Key milestones in this progression include social vocalization at 12 weeks, babbling at 8 months, and a vocabulary of 50–100 words at 21 months, word combinations at 2.5 years, and the use of complex sentences at 3 years [3, 7]. Falling behind these milestones in comparison to peers of the same age, gender, and cultural background is known as speech and language delay (SLD) [1, 6, 8, 9].

SLD is a common childhood developmental delay [2]. Among primary school students, around 5% are diagnosed with SLD, with global prevalence ranging from 3 to 20% [4]. Speech and language delay can be classified as primary or secondary. Primary SLD involves delays in both language reception and expression, with an unknown cause. Secondary SLD is linked to hearing or neurological impairments, developmental challenges, and behavioral or emotional difficulties [3, 10]. SLD can manifest as speech delay, language delay, or a combination, occurring together or separately. About 6% to 10% of children under six experience delayed language development, leading to consultations with pediatricians [3, 8, 11]. Similarly, speech delay often indicates various mental and physical conditions rather than a distinct diagnosis [12].

Untreated SLD can cause ongoing problems for 40–60% of children, increasing the risk of cognitive, emotional, behavioral, and social issues in adulthood [4, 13–15]. Children with this condition may struggle with independence and have a higher likelihood of psychiatric disorders later in life [16]. Compared to typically developing peers, teenagers with speech impairments show significant behavioral and emotional symptoms, with 35–50% requiring support [17].

In studies conducted in Eastern India, Iran, and Cuba, it was found that males had a higher prevalence of speech and language delay [1, 18, 19]. However, in Nepal, females exhibited a higher incidence SLD [11]. Family-related characteristics such as consanguinity(having close blood relations), low parental education, poor communication, monolingualism, and family history strongly correlate with SLD [2, 10, 11, 20–22]. Biological factors including seizure disorder, neonatal difficulties, birth hypoxia, oropharyngeal deformities, premature birth, prenatal alcohol consumption, and infectious diseases significantly predict SLD [2, 10, 20, 22–24]. Poor feeding habits was found to increase the risk of SLD in researches from Nepal, and Bangladesh [11, 23]. Additionally, excessive screen time, stressful family dynamics, and unfavorable home environments are associated with this condition [23, 25].

There are only a few studies available that focus on Developmental Delay (DD) in Ethiopia [26, 27]. Additionally, there is currently no documented data on the factors that contribute to this condition under study the country. The National Mental Health Strategy of Ethiopia recognizes the importance of screening programs and early interventions for addressing developmental issues in children [28]. However, the specific interventions and methods of implementation are not specified. To develop effective intervention strategies like family counseling, speech therapy, and play therapy [29], it is crucial to understand the underlying factors. Therefore, this study aims to identify the factors that determine speech and language delay among children aged 12 months to 12 years at Yekatit 12 Hospital in Addis Ababa, Ethiopia.

## Methods

### Study design, period and setting

From April 1 to April 28, 2023, an unmatched case–control study took place at Yekatit 12 Hospital in Addis Ababa, Ethiopia.

Yekatit 12 Hospital, established in 1915, is a referral center and teaching hospital in Ethiopia. It has more than 300 beds and comprises 16 different inpatient wards and 21 outpatient departments, including a specialized pediatrics outpatient department. What sets Yekatit 12 Hospital apart is that it is the only government hospital in the country that provides speech and language therapy services. The decision to select this hospital as the study site was based on its extensive range of speech and language therapy services available to the entire country. This allows for access to children from diverse socio-cultural backgrounds, ensuring a representative sample for the study.

### Study variables

#### Dependent variables

Speech and Language delay among children aged 12 months to 12 years old.

#### Independent variables

Socio-demographic characteristics (gender, paternal educational status, maternal educational status, father’s occupation, mother’s occupation, monthly income, number of siblings, child’s birth order, age of father at child’s birth, and age of mother at child’s birth).

Biological related characteristics (seizure disorder, hearing problems, low birth weight, preterm, types of birth, birth asphyxia, middle ear infection, oropharyngeal deformity).

Feeding related characteristics (feeding history, duration of exclusive breast feeding, complementary food introduction, history of thumb-sucking, history of pacifiers use).

Family related characteristics (type of family, number of family members, family history of SLD, mother and child separation, father leave home, and multilingual family).

Environmental related characteristics (history of recent trauma/stress, screen exposure time of the child, child-rearing behaviors, the time the caregiver started to talk with the child, reading stories/showing pictures, the father or other adult providing care, the frequency of father or other adult men providing care, and the usual method they keep the child occupies).

### Study participants

This study included 150 participants, with 50 confirmed cases of SLD and 100 control subjects with age ranging from 12 months to 12 years.

Due to limitations such as budget and time constraints, it was not feasible to recruit a larger sample size that would meet the standard requirements for statistical power. Consequently, achieving the desired levels of statistical power becomes challenging, even with a significant effect size and reduced variability. To ensure that the research question could still be adequately addressed, the authors made the decision to include children between the ages of 12 months and 12 years. This choice was made in order to overcome the limitations and obtain a sample size that is sufficient for the study.

### Inclusion and exclusion criteria

#### Inclusion criteria

The case group consisted of children who had previously been diagnosed with SLD by experienced speech-language pathologists at the hospital. The selection of these children involved consulting with speech-language pathologists and family caregivers, as well as conducting a comprehensive review of their medical records. This review aimed to exclude the presence of any other medical conditions, such as autism, cerebral palsy, hydrocephalus, or genetic disorders like Down syndrome. Additionally, these children met the eligibility criteria of being between 12 months and 12 years old, and they were undergoing speech therapy.

The control group consisted of individuals without any clinical diagnosis of SLD, aged between 12 months and 12 years, receiving follow-up treatment for unrelated conditions.

#### Exclusion criteria

Both the cases and controls were considered ineligible if they had medical records indicating the presence of autism, cerebral palsy, hydrocephalus, or genetic disorders such as Down syndrome. This determination was made after carefully reviewing the child's medical records and consulting with both their caregivers and speech and language pathologist.

### Sample size determination

To calculate the sample size for the unmatched case–control study, the Epi Info version 7.2.2.6 statistical software employed the double population proportion formula. The parameters included a 95% confidence interval, a 5% marginal error (d), and 80% power, with a case-to-control ratio of 1:2. Previous research indicated that exclusive breastfeeding duration and daily screen exposure exceeding 2 h were the most influential factors [7]. The largest sample size estimated among the risk factors was found to be 107. To account for a 10% non-respondent rate, the sample size was increased to 120, with 40 cases and 80 controls. Ultimately, a sample size of 50 cases and 100 controls was used for the study.

### Sampling technique

The sampling method involved selecting two consecutive controls for each case, using simple random sampling (lottery method) on the same day at the pediatric outpatient department (OPD).

### Data collection tools and procedures

The structured questionnaires used in the interviews were carefully developed by conducting a thorough review of relevant literature sources [2, 5, 7, 12, 15, 18, 30–40]. These questionnaires were divided into six sections, covering socio-demographic, biological, feeding, family, environmental related characteristics, and the child's current condition. The answer choices provided in the questionnaires were designed to be mutually exclusive, with additional instructions included for clarity. During the development process, the research objectives were kept in mind, and the characteristics of the target population were taken into consideration. Information obtained from children's medical records was also incorporated into the design of the questionnaires.

Initially, the questionnaires were created in English and then translated into Amharic by a skilled Amharic expert. To ensure accuracy and consistency, the translated tools were then translated back into English by proficient English experts.

To ensure the validity and reliability of the questionnaires, experts with diverse backgrounds, including a pediatrician, speech-language pathologists, therapist, public health expert, and English literature expert, assessed the face validity of the questionnaires. They carefully reviewed the questionnaires and provided feedback, leading to revisions that improved the overall quality of the questionnaires.

In order to assess the reliability of each domain covered in the questionnaires (Socio-Demographic characteristics, Biological Related Characteristics, Feeding Related Characteristics, Family Related Characteristics, and Environmental Related Characteristics), the Cronbach Alpha (α) values were calculated to determine internal consistency. The reliability coefficients for each domain were found to be 0.71, 0.73, 0.70, 0.81, and 0.78, respectively.

Three Bachelor of Science (BSc) nurses, supervised by a Master of Science (MSc) nurse, gathered data from parents or caregivers of both the case group and the control group.

### Data quality assurance

To ensure accuracy, a pretest was conducted on a 5% sample at Tikur Anbessa specialized Hospital (TASH) pediatrics OPD a week before the actual data collection started. Data collectors received one-day orientation/training, emphasizing proper questionnaire completion. Experts in the field assessed the questionnaire's consistency and equivalent in Amharic and English. Data double checking was performed, first by the collectors and then by the primary investigators who selected 10% of the completed data to assess consistency, errors, and data completeness.

### Data processing and analysis

To conduct data analysis, we utilized SPSS version 26 (Version 26.0. Armonk, NY: IBM Corp). For categorical variables, descriptive statistics such as frequencies and percentages were employed, while means and standard deviations were used for continuous variables. These measures allowed us to gain a comprehensive understanding of the data's characteristics through straightforward descriptive measures. The relationship between speech and language delay and related factors was assessed using Chi-square analysis, followed by logistic regression.

We utilized the chi-square test for two primary reasons. Firstly, in regression analysis, it is crucial to assume that the predictor variables are independent of each other. To verify this independence, we conducted the chi-square test. This test helped us determine whether the predictor variables were truly independent. Secondly, previous studies in Ethiopia primarily focused on general developmental delay without specifically addressing speech and language delay. Consequently, we encountered difficulty in identifying the most appropriate predictor variables for our analysis in our specific setting. To overcome this challenge, we conducted the chi-square test, which aided us in selecting the most suitable predictor variables.

In bivariate analysis, variables with a *P* < 0.25 were considered significant and subsequently included in a multiple multivariate logistic regression model. The variables that exhibited significant associations in a multiple multivariate logistic regression model were determined using the Adjusted Odds Ratio (AOR), along with a 95% confidence Interval (CI) and a *P* < 0.05. Furthermore, we conducted a multicollinearity test to assess the presence of multicollinearity among the variables and a Hosmer–Lemeshow goodness-of-fit test to evaluate the model's fit to the data. These tests were performed to ensure the integrity and dependability of the data analysis process.

## Result

### Socio-demographic characteristics of study participants

The study included 150 participants (50 cases, 100 controls) with a 100% response rate. Among cases, 70% had expressive delay, 14% had receptive delay, and 16% had both. Both cases and control had nearly equal mean age, 50.84 (SD = 34.43) and (51.42, SD = 32.21) respectively. Females had a higher prevalence of SLD (54%). The study found statistically significant associations between SLD and factors like family income, number of siblings, and mother's age at child's birth. Table 1

**Table 1:** Socio-Demographic characteristics for the study of determinants of speech and language delay among children 12 months to 12 years in Yekatit 12 Hospital, Addis Ababa, Ethiopia, 2023

Socio-demographic Characteristics	Cases(total = 50) *N* (%)	Controls ( total = 100) *N* (%)	*P*-Value
**Gender**			
Male	23 (46%)	54 (54%)	0.355
Female	27 (54%)	46 (46%)	
**Paternal educational status**			
Cannot read and write	6 (12%)	13 (13%)	0.470
Primary/Middle school	10 (20%)	24 (24%)	
High school	16 (32%)	19 (19%)	
Occupational training	5 (10%)	9 (9%)	
Diploma/Degree/other	13 (26%)	35 (35%)	
**Maternal educational Status**			
Cannot read and write	12 (24%)	19 (19%)	0.701
Primary/Middle school	12 (24%)	24 (24%)	
High school	12 (24%)	22 (22%)	
Occupational training	5 (10%)	7 (7%)	
Diploma/Degree/other	9 (18%)	28 (28%)	
**Father's occupation**			
Unemployed	14 (28%)	15 (15%)	0.570
Employed/Self- employed	36 (72%)	85 (85%)	
**Mother’s Occupation**			
House wife	37 (74%)	69 (69%)	0.526
Employed/Self- employed	13 (13%)	31 (31%)	
**Monthly income**			
≤ 5000	10 (20%)	36 (36%)	0.003
5001–10000	32 (64%)	36 (36%)	
> 10,000	8 (16%)	28 (28%)	
**Number of siblings**			
Zero	5 (5%)	14 (14%)	0.005
One	16 (32%)	37 (37%)	
Two	15 (30%)	21 (21%)	
Three	5 (10%)	14 (14%)	
Four	5 (10%)	7 (7%)	
Greater than four	4 (8%)	7 (7%)	
**Child’s birth order**			
First	21 (42%)	57 (57%)	0.305
Second	16 (32%)	24 (24%)	
Three	10 (20%)	13 (13%)	
Greater than three	3 (6%)	6 (6%)	
**Age of father at child's birth**			
≤ 20	0 (0%)	1 (1%)	0.069
21–30	8 (16%)	29 (29%)	
31–40	25 (50%)	53 (53%)	
> 40	17 (34%)	17 (17%)	
**Age of mother at child's birth**			
≤ 20	4 (8%)	1 (1%)	0.009
21–30	21 (42%)	63 (63%)	
31–40	25 (50%)	33 (33%)	
> 40	0 (0%)	3 (3%)	

*P* value < 0.05 considered as significant. *P* value was obtained by chi-square

### Biological related characteristics of study participants

Hearing problem, birth asphyxia, and oropharyngeal deformity were strongly associated with SLD among the nine considered biological factors. This association was statistically significant at p < 0.05. Table 2

**Table 2:** Biological factors for the study of determinants of speech and language delay among children 12 months to 12 years in Yekatit 12 Hospital, Addis Ababa, Ethiopia

Biological related characteristics	Cases (total = 50) *N* (%)	Controls (total = 100) *N* (%)	*P* = value
**Seizure disorder**			
Yes	7 (14%)	8(8%)	0.248
No	43 (86%)	92(92%)	
**Hearing problems**			
Yes	6 (12%)	1 (1%)	0.029
No	44 (88%)	99 (99%)	
**Low birth weight**			
Yes	9 (18%)	12 (12%)	0.318
No	41 (82%)	88 (88%)	
**Preterm**			
Yes	10 (20%)	13 (13%)	0.262
No	40 (80%)	87 (87%)	
**Types of birth**			
SVD	35 (70%)	69 (69%)	0.900
Cesarean section	15 (30%)	31 (31%)	
**Birth Asphyxia**			
Yes	9 (18%)	5 (5%)	0.010
No	41 (82%)	95 (95%)	
**Middle ear infection**			
Yes	5 (10%)	3 (3%)	0.072
No	45 (90%)	97 (97%)	
**Throat Infection**			
Yes	4 (8%)	7 (7%)	0.825
No	46 (92%)	93 (93%)	
**Oropharyngeal deformity**			
Yes	7 (14%)	2 (2%)	0.004
No	43 (86%)	98 (98%)	

*P* value < 0.05 considered as significant. *P* value was obtained by chi-square

### Feeding related characteristics of study participants

The study found that feeding history, which includes breast feeding, bottle feeding, and mixed feeding, was the only factor related to feeding that showed a statistically significant association with SLD. Table 3

**Table 3:** Feeding factors for the study of determinants of speech and language delay among children 12 months to 12 years in Yekatit 12 Hospital, Addis Ababa, Ethiopia, 2023

Feeding Factors	Cases (total = 50) *N* (%)	Control (total = 100) *N* (%)	*P*-value
**Feeding History**			
Breastfeeding	37 (74%)	94 (94%)	0.014
Bottle-fed	9 (18%)	3(3%)	
Mixed	4 (8%)	3(3%)	
**Duration of Exclusive breast feeding**			
Less than 6 months	7	9	0.380
6 months	6	19	
Greater than 6 months	28	69	
**Complementary food introduction time**			
Before 6 months	10 (20%)	8 (8%)	0.083
6 months	5 (5%)	8 (8%)	
After 6 months	35 (35%)	84 (84%)	
**History of thumb-sucking**			
Yes	20 (40%)	35 (35%)	0.549
No	30 (60%)	65 (65%)	
**History of pacifiers use**			
Yes	18 (36%)	27 (27%)	0.257
No	32 (64%)	73 (73%)	

*P* value < 0.05 considered as significant. *P* value was obtained by chi-square

### Family related characteristics of study participants

This study analyzed various family factors, including family type, size, history of SLD, mother–child separation, father's absence, and having a multilingual family. Among these, the number of family members and having a family history of SLD were significantly associated with speech and language delay. Table 4

**Table 4:** Family-based factors for the study of determinants of speech and language delay among children 12 months to 12 years in Yekatit 12 Hospital, Addis Ababa, Ethiopia, 2023

Family related factors	Cases (total = 50%) *N* (%)	Controls (total = 100) *N* (%)	*P*-value
**Type of family**			
Joint family	10 (20%)	24 (24%)	0.581
Nuclear Family	40 (40%)	76 (76%)	
**No of family members**			
Equal to or less than four	21 (42%)	59 (59%)	0.049
Greater than four	29 (58%)	41 (41%)	
**Family history SLD**			
Yes	11 (22%)	10 (10%)	0.046
No	39 (78%)	90 (90%)	
**Mother and child separation**			
Yes	13 (26%)	17 (17%)	0.194
No	37 (74%)	83 (83%)	
**Father leave home**			
Yes	36 (72%)	80 (80%)	0.270
No	14 (28%)	20 (20%)	
**Multilingual family**			
Yes	21 (42%)	29 (29%)	0.111
No	29 (58%)	71 (71%)	

*P* value < 0.05 considered as significant. *P* value was obtained by chi-square

### Environmental related characteristics of study participants

In terms of environmental factors analyzed in this study, it was found that only screen exposure time had a significant association with SLD. Table 5

**Table 5:** Environmental factors for the study of determinants of speech and language delay among children 12 months to 12 years in Yekatit 12 Hospital, Addis Ababa, Ethiopia, 2023

Environmental Related Factors	Cases (total = 50) *N* (%)	Controls (total = 100) *N* (%)	*P*-Value
**History of recent trauma or stress**			
Yes	6 (12%)	9 (%)	0.564
No	44 (88%)	91 (91%)	
**Screen exposure time of the child**			
0-2 h	10 (20%)	41 (41%)	0.010
≥ 2 h	40 (80%)	59 (59%)	
**Child-rearing behaviors**			
Rude	1 (2%)	2 (2%)	0.523
Strict	11 (22%)	33 (33%)	
Gentle/friendly	30 (60%)	54 (54%)	
Permissive	8 (16%)	11 (11%)	
**The time the caregiver started to talk with the child**			
0–3 months	11 (22%)	18 (18%)	0.227
3–9 months	11 (22%)	38 (38%)	
9–15 months	17 (34%)	30 (60%)	
When he/she was old enough to understand	11 (22%)	14 (14%)	
**Reading stories or showing pictures**			
Hardly ever	23 (46%)	42 (42%)	0.523
Once or twice a month	5 (10%)	8 (8%)	
At least once a week	5 (10%)	20 (20%)	
At least 3 times a week	8 (16%)	18 (18%)	
At least 5 times a week	9 (18%)	12 (12%)	
**The father or other adult men providing care**			
Yes	49 (98%)	95 (95%)	0.121
No	1 (2%)	5 (5%)	
**The frequency of father or other adult men providing care**			
At least once a month	5 (10%)	10 (10%)	0.970
At least once a week	5 (10%)	10 (10%)	
At least 3 to 4 times a week	15 (30%)	32 (32%)	
Every day	25 (50%)	48 (48%)	
**The usual method they keep the child occupied**			
Give him/her something to eat	1 (2%)	5 (5%)	0.605
Offer him/her a toy	17 (34%)	25 (25%)	
Put him/her to nap	9 (18%)	21(21%)	
Encourage him/her to keep himself/herself busy	11 (22%)	29 (29%)	
Play with him/her	12 (24%)	20 (20%)	

*P* value < 0.05 considered as significant. *P* value was obtained by chi-square

### Bivariate and multivariable logistic regression analysis results of the study

In this unmatched case–control study, a various independent variables was examined to determine their impact on SLD. Bivariate logistic regression analysis yielded insightful findings, indicating a significant association at *P* < 0.25 between speech and language delay and several variables. These include factors such as father's occupation, place of residence, father's age exceeding forty, young maternal age, hearing impairments, birth complications (such as asphyxia), middle ear infections, oropharyngeal deformities, bottle-feeding practices, introduction of complementary foods prior to six months, limited family size of four or less, family history of SLD, instances of mother–child separation, multilingual familial backgrounds, extended screen time surpassing two hours, as well as certain child-rearing behaviors associated with SLD. The results of the multivariable analysis revealed that birth asphyxia, feeding history, mother–child separation, multilingual family, and screen time more than two hours were significant determinants of SLD.

Children who experienced birth asphyxia were about 4.58 times to suffer from SLD compared to children who had no birth asphyxia [AOR = 4.58, 95CI (1.23–16.99)]. The odds of SLD were 4.54 times higher in bottle-fed children compared to breastfed children [AOR = 4.54, 95CI (1.29–16.04)]. Children who were separated from their mother were 2.6 times more likely to have SLD compared to those who had not to separate from their mother [AOR = 2.6, 95CI (1.05–6.43)]. Additionally, children from multilingual families had a 2.31 times higher risk of developing SLD compared to those from non-multilingual families [AOR = 2.31, 95CI (1.03–5.18)]. Furthermore, children who spent more than two hours viewing screens (television, mobile devices, or laptops) were at a higher risk of developing SLD, with odds of 3.06 [AOR = 3.06, 95CI (1.29–7.28)]. Table 6

**Table 6:** Bivariate and multivariable analysis for the study of determinants of speech and language delay among children 12 months to 12 years in Yekatit 12 Hospital, Addis Ababa, Ethiopia, 2023

Variables	Cases (total = 50) *N* (%)	Controls (total = 100) *N* (%)	COR with 95%CI	AOR with 95%CI
**Father’s Occupation**				
Unemployed	14(28%)	15(15%)	2.20(0.96–5.03)	1.63(0.62, 4.28)
Employed/self-employed	36(72%)	85(85%)	1	1
**Place of residence**				
Urban	41(82%)	89(89%)	0.56(0.22–1.46)	0.64(0.18, 2.28)
Rural	9(18%)	11(11%)	1	1
**Age of the father**				
≤ 20	0(0%)	1(1%)	0.00	0.00
21–30	8(16%)	29(29%)	1	1
31–40	25(50%)	53(53%)	1.71(0.68–4.27)	3.13(0.79–12.45)
> 40	17(34%)	17(17%)	3.62(1.29–10.17)	7.34(1.60–33.55)
**Age of the mother**				
≤ 20	4(8%)	1(1%)	4.92(1.08–22.38)	5.75(0.84–39.48)
21–30	21(42%)	63(63%)	1	1
31–40	25(50%)	33(33%)	2.21(1.07–4.57)	2.65(1.15–3.67)
> 40	0(0%)	3(3%)	0.00	0.00
**Hearing problems**				
Yes	6(12%)	1(1%)	5.44(1.02–29.136)	4.04(0.62–26.41)
No	44(88%)	99(99%)	1	1
**Birth asphyxia**				
Yes	9(18%)	5(5%)	4.17(1.32–13.21)	4.58(1.23–16.99)*
No	41(82%)	95(95%)	1	1
**Middle ear infection**				
Yes	5(10%)	3(3%)	3.59(0.82–15.69)	0.93(0.10–8.67)
No	45(90%)	97(97%)	1	1
**Oropharyngeal deformity**				
Yes	7(14%)	2(2%)	7.98(1.59–39.98)	2.75(0.37–20.49)
No	43(86%)	98(98%)	1	1
**Feeding history**				
Breastfeeding	37(74%)	94(94%)	1	1
Bottle-fed	9(18%)	3(3%)	4.50(1.41–14.35)	4.54(1.29–16.04)*
Mixed	4(8%)	3(3%)	2.50(0.68–9.16)	3.75(0.87–16.12)
**Complementary food**				
Before 6 months	10(20%)	8(8%)	0.34(0.12–0.93)	0.61(0.17–2.22)
6 months	5(10%)	8(8%)	1	1
After 6 months	35(72%)	84(84%)	0.50(0.18–2.14)	1.20(0.21–7.04)
**Number of family members**				
Equal to or less than four	21(42%)	59(59%)	0.50(0.25–1.00))	0.76(0.32–1.90)
Greater than four	29(58%)	41(41%)	1	1
**Family history of Speech and language delay**				
Yes	11(22%)	10(10%)	2.34(1.00–6.47)	1.60(0.51–5.05)
No	39(78%)	90(90%)	1	1
**Mother–child Separation**				
Yes	13(26%)	17(17%)	1.72(0.76–3.89)	2.60(1.05–6.43)*
No	37(74%)	83(83%)	1	1
**Multilingual family**				
Yes	21(42%)	29(29%)	1.77(0.87–3.60)	2.31(1.03–5.18)*
No	29(58%)	71(71%)	1	1
**Screen time**				
Less than two	10(20%)	41(41%)	1	1
Greater to or equal to two	40(80%)	59(59%)	2.78(1.25–6.18)	3.06(1.29–7.28)*
**Child rearing behavior**				
Rude	1(2%)	2(2%)	0.69(0.05–8.96)	0.51(0.02–13.74)
Strict	11(22%)	33(33%)	0.46(0.15–1.43)	0.40(0.09–1.72)
Gentle/friendly	30(60%)	54(54%)	0.76(0.28–2.11)	1.01(0.26–4.02)
Permissive	8(16%)	11(11%)	1	1

Key: 1 = Reference, * = statistically significant with *P*-value < 0.05

## Discussion

Extensive research has been conducted on SLD, yet there remains a dearth of comprehensive studies focusing on the determinants of SLD, specifically in developing nations like Ethiopia. As a result, this study aimed to identify the determinants of speech and language delay among children whose age ranges from 12 months to 12 years, with the ultimate goal of informing the development of effective interventional strategies.

Despite the increased prevalence of SLD observed among males, similar to numerous other studies [2, 7, 8, 10, 11, 21, 23, 41], in this study no significant association was found between gender and SLD. In contrast, a couple of studies have indicated that males have a twofold higher likelihood of being diagnosed with SLD [41, 42]. It is evident that the socioeconomic status of a family can contribute to a child's development of various health risks, and SLD is no exception to this phenomenon [43]. Moreover, a study has revealed that there is a direct association between the level of family income and the quality of early child care, which significantly influences the child's speech and language development in later years [44]. However, unlike previous studies [11, 45–47], the present study could not demonstrate any statistical association between SLD and the socioeconomic status of the family.

Numerous studies have consistently shown that children with a familial background of SLD face a substantially higher risk, up to nine times, experiencing similar delay themselves [1, 2, 14, 23, 35, 41, 48]. Nonetheless, in our particular study, we were unable to establish a significant association between family history and SLD (AOR = 1.60; CI = 0.51–5.05; *P* > 0.05). Interestingly, other studies [2, 14, 49] have encountered similar challenges when attempting to establish a clear statistical association between family history and SLD, potentially due to the study's utilization of a hospital-based design and similarly constrained sample size.

The quality of the relationship between the child and their maternal figures plays a significant role in language and cognitive development [50]. In light of this, this study demonstrated that the separation of a mother and child determines the occurrence of SLD significantly (AOR = 2.60; CI = 1.05–6.43; *P* < 0.05). This profound finding resonates with corroborative studies executed in both Korea and Pakistan, which have also detected a statistically significant association between mother–child separation and SLD [48, 51]. This association may be attributed to the reduced levels of care, companionship, and inadvertent negligence that materialize when the mother is absent. On the contrary, research conducted in Bangladesh failed to furnish any compelling evidence supporting a significant association within this specific sphere [23].Nevertheless, it is important to note that the ongoing trend of more women actively participating in the workforce outside the home, similar to men, it has inadvertently led to a situation where children spend more time under the care of others, creating a greater gap between mothers and children. Consequently, children are becoming increasingly absorbed in technological screens, such as televisions and mobile gaming devices.

Multiple studies, including ours, have discovered a statistically significant association between prolonged screen time exposure and the onset of SLD [2, 7, 14, 23, 51]. Specifically, children who spend extended periods engaged with screens are nearly three times more likely to experience SLD compared to those with limited exposure of less than two hours [7, 23, 51]. Remarkably, our study aligns with these findings, revealing an odds ratio of 3.06 at *P* < 0.05. Engaging children in linguistic diversity during their learning experiences is vital. This is because they may encounter difficulties in transferring information acquired from screen-based devices [52]. It is worth noting that the consistency in results among various studies, including ours, may be attributed to similarities in study design, and participant characteristics.

Children who are raised in a bilingual environment commonly display a phenomenon known as linguistic mixing, whereby they blend elements from both languages they are exposed to. As their language skills progress, this mixing diminishes. However, we have observed that in some cases, when children mix the two languages excessively, it can lead to a perceived delay in speech development [4, 53]. In our research, we discovered that children coming from multilingual families had an elevated likelihood of experiencing SLD at odds ratio of 2.31 at statistically significant *p*-value of less than 0.05. Interestingly, this finding aligns with a study conducted in India [10], highlighting the influence of factors such as study design and the age range of participating children, which spanned from 1 to 12 years old.

This study has uncovered a significant association between birth asphyxia and SLD. It was found that children who experienced birth asphyxia were 4. 58 times more likely to encounter SLD. This finding is consistent with a study conducted in India [10] and another study in Bangladesh, which reported odds of delay at 4.72 [50]. This association can potentially be explained by the linguistic challenges that may arise from neonatal hypoxic-ischemic encephalopathy, a brain injury caused by the disruption of blood flow during birth [54].

Bottle-feeding has been associated with a higher risk of developing ear infections, which can subsequently lead to a decrease in hearing ability and speech and language difficulties [55]. In this study, it was found that children who were given bottle-fed were 4.54 times more likely to experience SLD compared to those who were breast-fed. On the other hand, breast feeding acts as a protective factor against SLD by promoting normal development of the oro-facial structures and enhancing coordination among the muscles of the tongue, mouth, lips, and jaw [55]. Additionally, it worth noting that breast milk provides optimal nutrition for neurodevelopment, fulfilling the nutritional requirements of a child until the age of six months [56].

As a limitation in the current study, anthropometric measurements were not conducted on the children in order to determine whether nutritional status determines language and speech development. Thus, the next researcher can establish if nutritional status determines speech and language development. This study may have biased sample selection as it was conducted in a hospital-based setting. So, the next researcher can conduct a community-based study and explore whether mothers can identify SDL in the early years of their children in order to start early therapy. Because the exposure status was measured retrospectively, there could be recall bias.

## Conclusions

SLD stands as one of the most prevalent developmental delay (DD). If left untreated, it can engender a plethora of complications encompassing social, cognitive, and behavioral domains. Multiple determinants are believed to underlie the manifestation of SLD. Remarkably, this study has unearthed highly notable and statistically significant determinants that give rise to SLD in Ethiopia, including birth asphyxia, reliance on bottle-feeding, the unfortunate circumstance of mother–child separation, exposure to multiple languages, as well as excessive screen time surpassing two hours per day.

## Data Availability

The datasets used and analyzed in the current study are available upon reasonable request from corresponding author.

## Abbreviations

AOR: Adjusted Odds Ratio
CI: Confidence Interval
OPD: Outpatient Department
TASH: Tikur Anbessa specialized Hospital
SLD: Speech and Language Delay
